# Routine evaluation of left ventricular diastolic function by cardiovascular magnetic resonance: A practical approach

**DOI:** 10.1186/1532-429X-10-36

**Published:** 2008-07-08

**Authors:** Vikas K Rathi, Mark Doyle, June Yamrozik, Ronald B Williams, Ketheswaram Caruppannan, Craig Truman, Diane Vido, Robert WW Biederman

**Affiliations:** 1 Division of Cardiology, Allegheny General Hospital, Drexel University College of Medicine, 320 E North Avenue, Pittsburgh, PA- 15212, USA

## Abstract

**Background:**

Cardiovascular magnetic resonance (CMR) has excellent capabilities to assess ventricular systolic function. Current clinical scenarios warrant routine evaluation of ventricular diastolic function for complete evaluation, especially in congestive heart failure patients. To our knowledge, no systematic assessment of diastolic function over a range of lusitropy has been performed using CMR.

**Methods and Results:**

Left ventricular diastolic function was assessed in 31 subjects (10 controls) who underwent CMR and compared with Transthoracic echocardiogram (TTE) evaluation of mitral valve (MV) and pulmonary vein (PV) blood flow. Blood flow in the MV and PV were successfully imaged by CMR for all cases (31/31,100%) while TTE evaluated flow in all MV (31/31,100%) but only 21/31 PV (68%) cases. Velocities of MV flow (E and A) measured by CMR correlated well with TTE (r = 0.81, p < 0.001), but demonstrated a systematic underestimation by CMR compared to TTE (slope = 0.77). Bland-Altman analysis of the E:A ratio and deceleration time (DT) calculated from each modality showed excellent agreement (bias -0.29, and -10.3 ms for E:A and DT, respectively). When assessing morphology using TTE, CMR correctly identified patients as having normal or abnormal inflow conditions.

**Conclusion:**

We have shown that there is homology between CMR and TTE for the assessment of diastolic inflow over a wide range of conditions, including normal, impaired relaxation and restrictive. There is excellent agreement of quantitative velocity measurements between CMR and TTE. Diastolic blood flow assessment by CMR can be performed in a single scan, with times ranging from 20 sec to 3 min, and we show that there is good indication for applying CMR to assess diastolic conditions, either as an adjunctive test when evaluating systolic function, or even as a primary test when TTE data cannot be obtained.

## Background

Congestive heart failure (CHF) is the third most prevalent form of heart disease with as many as 5 million patients currently under treatment in United States [1]. Approximately 40–50% of these have normal or near normal systolic function and therefore left ventricular (LV) diastolic dysfunction is the primary cause of CHF in such patients [2-5]. Despite the high prevalence of diastolic dysfunction, there have been fewer advances in diagnosis, which has probably stalled the understanding of its underlying pathophysiology. Currently, two biomechanical indices are used: LV relaxation and compliance [1,6], which are assessed via measurements of the mitral valve (MV) diastolic flow data with corroborating pulmonary vein (PV) velocity data.

Use of angiography for routine assessment of diastolic function by measuring the LV pressure decline over time (-*d*p/*d*t) as a function of LV relaxation has fallen out of favor, as non-invasive methodologies have become widely available. Currently, TTE is almost exclusively used to assess diastolic function, but in this application it is used to assess flow as opposed to volumetric conditions. Pulsed wave Doppler is applied to assess early MV diastolic filling velocity (Evel), late atrial systolic filling velocity (Avel), and deceleration time (DT). Similarly, the PV systolic 'S' wave and diastolic 'D' wave velocities have been used to confirm MV diastolic disturbances [7,8]. These diastolic indices have also been used to estimate the pulmonary wedge pressure, thus further enhancing their clinical utility [9]. Despite these advances, TTE has important disadvantages, including limited field of view, dependence on sample volume location, cosine θ errors relative to the flow direction and an inability to image approximately 15–20% of patients.

Over the last decade, CMR has been widely accepted as the "gold standard" for the assessment of systolic function due to its high spatial and temporal resolution, excellent image quality, lack of geometric assumptions and the ability to interrogate flow in 3D using phase contrast imaging (PC) [10,11]. The CMR-PC approach has been validated *in vitro *and *in vivo *and importantly, can be used to assess velocity in a 3D manner [12,13]. As the awareness in diastolic heart failure is increasing, there is increasing demand to identify a robust technique to provide accurate and comprehensive clinical and research diastolic function data. We hypothesize that assessment of diastolic function by examination of MV and PV flow conditions is clinically feasible using 3D CMR-PC imaging. While patients may not be primarily referred for diastolic function evaluation, in this manuscript we establish the validity of CMR diastolic function assessment by demonstrating its ability to characterize a range of diastolic impairments. This will likely become an important adjunctive test, adding to the comprehensive nature of the LV function evaluation.

### Study Design

A total of 31 subjects (21 male and 10 female) were studied, which included 21 patients with mean age of 60 ± 14 yrs. and 10 controls with mean age of 33 ± 9 yrs. Patients were recruited if they had prior evidence of diastolic dysfunction, either due to hypertensive heart disease, ischemic heart disease, cardiomyopathy or pericardial disease. All subjects were in normal sinus rhythm at the time of study. The study was approved by the institutional review board of Allegheny General Hospital and all subjects provided informed consent. The study design was a double blind evaluation of diastolic indices of MV and PV flow. *In vitro *validation of CMR-PC data was conducted by measuring pulsatile flow through a straight pipe generated by a pump under computer control (Shelly, Vancouver Canada). The mitral valve Evel, Avel, DT and pulmonary vein S and D wave velocities obtained by CMR were compared with similar indices obtained using TTE pulsed Doppler. By design, all subjects underwent TTE evaluation by a research echocardiographer (CT) using American Society of Echocardiography (ASE) guidelines within two hours of the CMR examination [14]. The Echo technologist and the physician were blinded to CMR results and the TTE and CMR data sets were evaluated separately by two independent physicians (RB, VR).

## Methods

### a) CMR Technique

**Figure 1:**
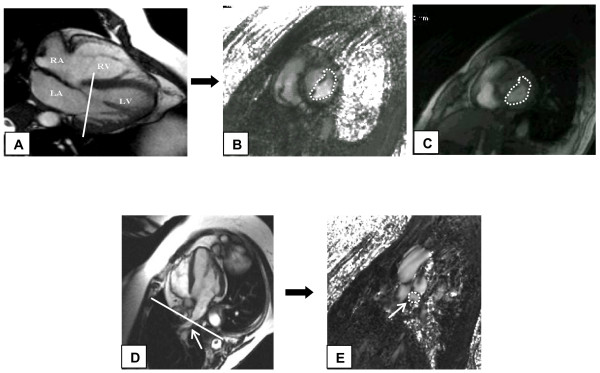
Image acquisition of velocity encoded (VENC) cine cardiovascular magnetic resonance imaging (CMR) at the level of mitral valve (MV). A) Cine 4 chamber image demonstrating the prescription of slice at the level of the tips of the MV leaflets. B) VENC CMR short axis image at the level of MV leaflet tips. Contour is drawn on the leaflets as shown including the whole cross-section of the mitral inflow. C) Magnitude image corresponding to the phase image. Note that the entire MV plane is interrogated as compared to the ice-pick view of conventional transthoracic echocardiography (TTE) imaging. D) Cine 4 chamber image demonstrating the position of slice 1 cm inside the right superior pulmonary vein (arrow) entrance into the left atrium. E) VENC CMR cross-section image of the right superior pulmonary vein with contours (arrow).

All subjects underwent PC imaging using a GE CV/i 1.5 T MRI scanner (Milwaukee, WI). Subjects were imaged in the supine position and signal reception was accomplished using a 4-channel phased-array cardiac coil. The ECG signal was used to trigger the acquisition. Localization scout images were acquired and used to identify the LV in the long axis four-chamber views. Cine images were acquired using Fast Imaging Employing STeady-state Acquisition (FIESTA) to permit visualization of the tips of the mitral valve leaflets. The right superior pulmonary vein was identified and imaged 1 cm proximal to the ostium. All PC data sets were acquired with velocity encoding in three orthogonal directions and velocity sensitivity set at 200 cm/s for each direction. The maximum velocity was calculated using the vector sum of the three individual velocity directions. To capture cross-sectional MV flow, an imaging plane was planned parallel to the mitral annular plane at the level of the mitral leaflet tips (Figure 1). The PV PC images were planned perpendicular to the pulmonary vein, and 1 cm inside the junction of the pulmonary vein with the left atrium (Figure 1). The PC images were acquired using retrospective ECG gating under free breathing conditions, with the following average parameters: slice thickness 7 mm, field of view 38 cm2, matrix 256 × 192, repetition time 7.0 ms, echo time 3.2 ms, flip angle 20° and 2 averages. Complete coverage of the cardiac cycle was accomplished using view-sharing to acquire 60 cardiac phases per cycle, resulting in a high temporal resolution (17–23 ms depending on heart rate). The segmentation factor of 4 was used for CMR-PC 3D acquisition, which resulted in fundamental resolution of 48 ms to 56 ms depending on the repetition time.

CMR is well established for steady flow assessment, but its validity in pulsatile flow is not as well established [15-17]. We used a phantom with pulsatile flow through a straight-pipe, which was imaged in a transverse manner using similar PC parameters as described earlier in this manuscript. Pulsatile flow was generated by a CV pump system (Shelly Medical Imaging, Vancouver, Canada) and fluid with a viscosity similar to blood was used (60% glycerin, 40% distilled water). Flow was generated by pulsatile flow pump under computer control with average flow rate at values ranging from 50 to 300 ml/cycle in 50 ml/cycle increments. A mean of 3 peak velocities at each flow rate was used.

**Figure 2:**
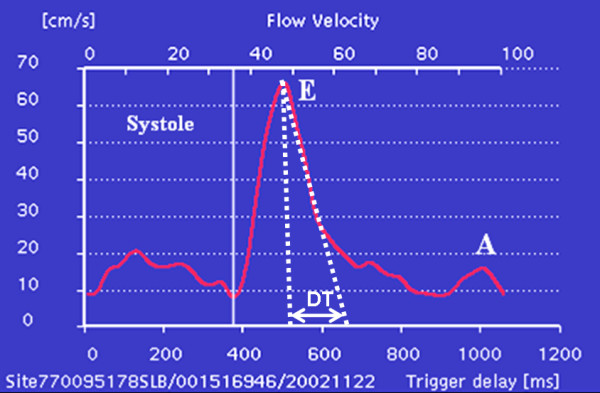
Demonstration of obtaining DT from the CMR mitral valve flow data. On the CMR flow curve a vertical line was drawn from the peak of E wave to intersect the baseline, which displayed time delay at which the E wave peak occurred. A second line was drawn from the peak of the E wave following the downslope intersecting the baseline, which gave the time delay of the E wave downslope. The time delay of the peak E wave was subtracted from the time delay of the E wave downslope intersecting the baseline to calculate the DT.

Images were analyzed offline on semi-automatic Medis CV flow 3.1 version program (Medis, The Netherlands). Regions of interest were manually drawn on one frame to encircle the entire cross section of MV leaflets or PV on the short axis images and propagated using a semi-automated contouring mode with manual override, yielding velocity *vs*. time graphs characterizing mitral diastolic E and A waves and pulmonary diastolic S and D waves. The mean of maximum velocity obtained for both MV and PV was recorded. The absolute maximal velocities were corrected for offsets by subtracting the residual velocity registered in a static background region drawn in the chest wall. The DT was calculated using the method described by Appleton CP et al [18]. On the CMR flow curve, a vertical line was drawn from the peak of E wave to intersect the baseline which displayed time delay at which the E wave peak occurred. A second line was drawn from the peak of the E wave following the downslope intersecting the baseline which gave the time delay of the E wave downslope. The time delay of the peak E wave was subtracted from the time delay of the E wave downslope intersecting the baseline to calculate the DT (Figure 2).

### b) Echocardiography Technique

The transthoracic echocardiogram was performed on a Philips Sonos 5500 with 3.0 MHz probe (Andover, MA). The exam was performed with the subject supine in the left lateral position. In the four-chamber view, the MV inflow was localized and the Doppler sample volume was placed at the level of the tip of the MV leaflets and the pulsed wave Doppler was recorded under free breathing conditions. Similarly, for the PV flow the sample volume was placed 1 cm inside the right superior pulmonary vein. The right upper pulmonary vein was chosen due to its ease of location by the echocardiographer and to provide continuity between CMR and echocardiographic data. Data were post-processed offline following ASE guidelines for the calculation of maximum E and A velocities, DT and pulmonary vein S and D velocities. A mean of 4 consecutive data sets was used for data analysis. Absolute velocity was converted to pressure gradient using modified Bernoulli equation (ΔP = 4 V^2^) [19,20].

### Statistics

After passing the, Kolmogorov-Smirnov normality test to assure parametric nature of acquired data, the student's *t *test was used to analyze paired data and regression analysis was used for correlation of CMR with TTE for corresponding variables. A p value of < 0.05 was taken as significant. Bland Altman analysis was performed to assess agreement between the CMR and TTE.

## Results

There was no significant difference in the summed acquisition and offline processing time for either technique (CMR mean time = 15 ± 2 min vs. TTE mean time = 13 ± 1 min). All 31/31 (100%) of MV and PV were imaged using CMR-PC in a mean duration of 2 ± 0.5 min each. All MV's were imaged by TTE. However, only 21 out of 31 (68%) PV were imaged by TTE and were not included in further analysis.

**Figure 3:**
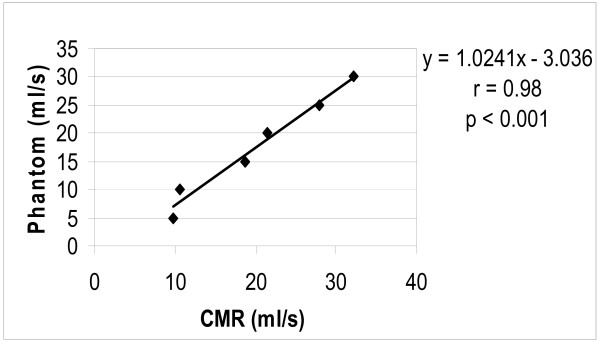
Correlation between CMR VENC derived stroke volume and pump phantom stroke volume *in-vitro*.

**Figure 4:**
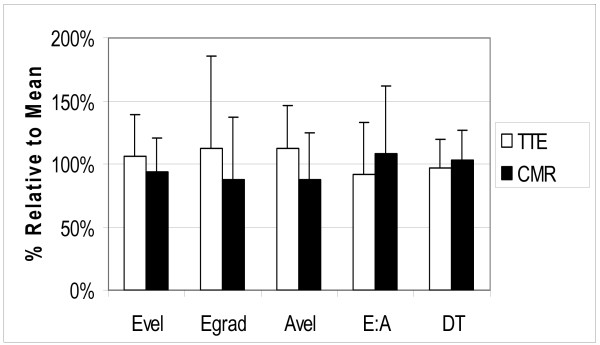
Comparison of normalized TTE and CMR parameters: plotted are the average values (expressed as a percentage of the mean) for each parameter, where Evel is maximal "E" velocity, Egrad is the gradient derived from Evel, Avel is maximal A velocity, E:A is the ratio of maximal E and A velocities and DT is deceleration time.

**Figure 5:**
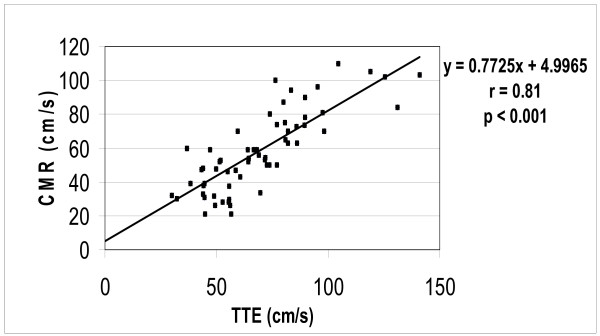
Correlation between CMR and TTE for maximal E and A velocities.

**Figure 6:**
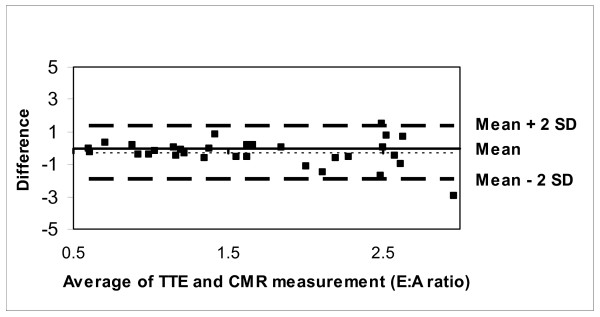
Bland-Altman plot demonstrating excellent agreement between CMR and TTE for E:A ratio.

**Figure 7:**
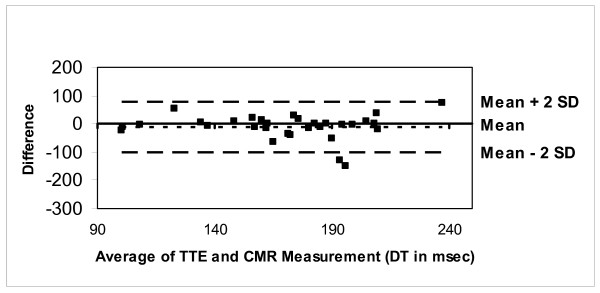
Bland-Altman plot demonstrating excellent agreement between CMR and TTE for deceleration time.

The CMR-PC data obtained from the flow phantom was planimetered to measure the stroke volume, which correlated well with the pump settings (r = 0.98, p < 0.001) (Figure 3). Similarly, *in vivo *measurements of the mitral valve peak Evel and Avel velocities by CMR-PC were slightly lower than TTE but correlated well (mean CMR peak Evel= 70.66 ± 20.3 cm/s, TTE Evel= 79.66 ± 24.7 cm/s; CMR Avel= 45.16 ± 18.8 cm/s, TTE Avel= 57.36 ± 17.7 cm/s; r = 0.81, p < 0.001) (Figures 4 and 5). Further, the Evel and Avel gradients measured by CMR and TTE were highly correlated (r = 0.80, p < 0.001). Importantly, the E:A ratio and the DT measured by CMR (1.8 ± 0.8 and 175 ± 40.7 ms respectively) and TTE (1.5 ± 0.7 and 165 ± 39.2 ms respectively) were not different when assessed using a paired t-test. Bland-Altman analyses demonstrated strong agreement for E:A ratio (bias= -0.29 cm/s) and DT (bias= -10.359 ms) between CMR and TTE (Figures 6 and 7).

**Figure 8:**
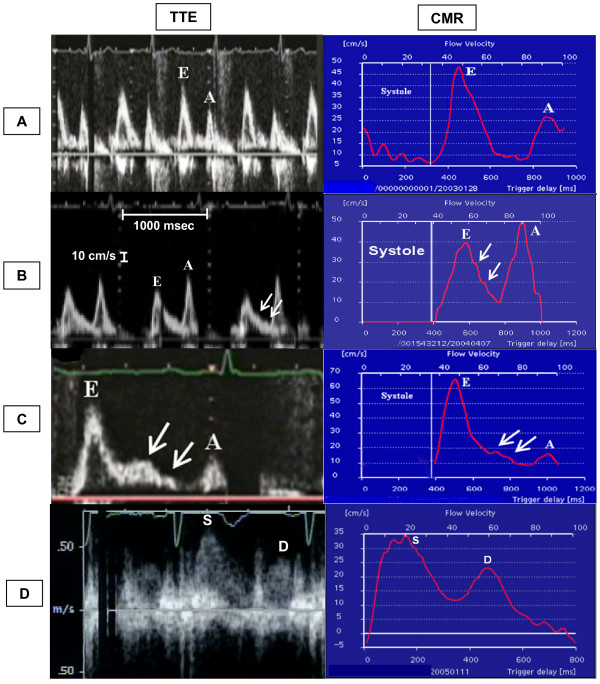
Morphological representation of velocities obtained by TTE and CMR. Note comparability between TTE and CMR representation of E and A waves with respect to their magnitude and temporal presentation in diastole. The x axis of the TTE panel is labeled on panel B figure and was calibrated to 1000 msec between two vertical dotted lines for all the patients and the y axis is represented by the smaller distance between the dots of the vertical dotted lines and was calibrated to 10 cm/sec for all patients. The y axis calibration for pulmonary vein flow (panel D) was different from the transmitral flow and is shown on the image; however, the x axis calibration was similar. Panel A demonstrate normal E:A ratio and deceleration time (DT). Panel B shows impaired relaxation with prolonged DT and a high A wave. Panel C demonstrates a restrictive pattern with short DT and an elevated E:A ratio. Note diastasis points (arrows) illustrating the similarities in depiction of flow features. In panel D the flow velocity profile obtained by CMR of right superior pulmonary vein is similar to that obtained with TTE (S and D are the systolic and diastolic waves of the pulmonary vein).

The 31 subjects demonstrated diverse diastolic physiology: 11 impaired relaxation, 3 restrictive, 2 pseudonormalized, 3 EA fusions and the remainder were assessed as normal using TTE MV flow data. Morphology was assessed by the standard classification of the graphical representation of flow velocities. In each case, the CMR representation of diastolic flow velocities was similar to the TTE, allowing identical morphologic classifications (Figure 8).

## Discussion

A comprehensive clinical evaluation of ventricular function for CHF requires assessment of both LV systolic and diastolic function. Conventionally, echocardiographic Doppler measurements of MV flow conditions are used to assess diastolic function. However, CMR-PC imaging allows quantitative assessment of blood velocity, with the advantage that the tomographic plane of interest can be positioned optimally, permitting measurement in 3D space compared to the "ice pick" assessment of flow allowed by echocardiography.

Flow velocity based diagnosis of diastolic function requires an accurate representation of changes throughout the diastolic time of mitral inflow. Indeed, our most important finding was that morphologically the CMR data had 100% correlation with the TTE data, and further we have demonstrated its practical utility. Additionally, quantitative CMR measurement of variables including Evel, Avel, E:A ratio and deceleration time were highly correlated with TTE measurements. Despite excellent correlations, Evel and Avel measured by CMR were systematically lower compared to TTE. However, the CMR acquisition of velocities in a pulsatile flow phantom accurately correlated with pump determined flow values. This may reflect the differences in the nature of acquisition by each technique. TTE obtains data within each cardiac cycle and is thus influenced by changes that occur over short time scales, whereas CMR data are effectively averaged over several cycles, thus damping sensitivity to intra-cycle variation. Importantly, this did not result in any misclassification of diastolic flow abnormalities between modalities. As CMR progresses towards real-time modes, these areas of difference are expected to diminish.

Our results are in concordance with the previous, but limited, studies [18,19]. A comparative study between CMR and TTE for diastolic function evaluation was performed by Hartiala et al in 1993. They studied 10 normal individuals and achieved modest correlations between TTE and CMR-PC (Evel r = 0.68 and Avel r = 0.83) [21]. The MV and PV were imaged by CMR-PC using both 2D and 3D velocity encoding were both lower than the TTE measured velocities. The study was performed on early generation scanners and since then the imaging technique has evolved to allow better temporal resolution and faster imaging. Recently, powerful gradients and faster computing power has led to shorter echo time and repetition time, which has led to better results. These technical improvements have also led to real-time CMR applications.

A similar approach was used by Karwatowski et al in 1995, studying 19 patients with known coronary artery disease [22]. The technique increased the temporal resolution to 35 ms by using in-plane velocity encoding on horizontal long axis view. The study was not designed to correlate diastolic patterns but showed that CMR and TTE Evel values were similar, but Avel values were slightly lower by CMR. Recently, Lin SJ et al compared mitral valve area using pressure half time assessed independently by CMR-PC and echocardiography methods [23]. They obtained Evel and Avel velocities similar to echocardiography (r = 0.81 and r = 0.89, respectively). Although this study was not designed to evaluate diastolic function, it does show the feasibility of clinically employing CMR-PC acquisitions.

We imaged MV and PV in the short axis orientation, perpendicular to the major direction of flow, and using 3D encoding of velocity, allowing interrogation of the complete cross-section of the plane to comprehensively assess diastolic flow, irrespective of the location and vector. The velocity window of 200 cm/s was used to avoid aliasing of the Evel and was not significantly different to values used in prior studies. Pixel by pixel evaluation of 3D velocity data obtained by CMR revealed that most peak velocities reside in the anterolateral or inferoseptal location away from the center of the mitral valve where we typically sample by TTE. This finding should be confirmed in larger series of patients to give us a direction into newer insights of diastolic mitral flow evaluation.

In summary, our study was conducted to establish the feasibility of routinely performing evaluation of MV flow to assess diastolic function using CMR. We were able to obtain clinical variables including E:A ratios and deceleration times, which were in agreement with those obtained using TTE.

### Limitations

The diastolic flow indices are limited by the pressure gradients, heart rate and hydration status of the patient. This source of variability is not technique dependent, and standard precautions were observed to minimize these influences. To obviate this limitation, intramyocardial properties ideally should be assessed, but this investigation is beyond the scope of the work presented here. While a diastolic strain analysis can be performed, this too is not generally considered clinically feasible due to the protracted post-processing required and the inability to compare directly with clinical assessments obtained using echocardiography.

Non-breathhold imaging was performed for CMR-PC. As rapid, sparse sampling techniques mature, such as BRISK (Block Regional Interpolation of Segmented k-space), the breathholds can be applied to PC imaging thus improving signal to noise and reducing variability due to respiratory effects and slice selection [24,25]. Post-processing, although is rapid for CMR, bus is not as convenient to perform when compared with routine pulsed wave Doppler analysis. As technical modifications progress, this limitation will diminish, as near real time acquisition and processing options will be available in CMR.

## Conclusion

This study highlights certain key points for the LV diastolic function assessment by CMR. Firstly, it is clinically feasible to accurately obtain the LV diastolic flow data and systolic function data in a single examination, wherein current clinical practice application of CMR is largely limited to systolic function evaluation. Secondly, the image quality, patient tolerability, ease and time to obtain the data match the TTE examination, and only adding an average of 4 minutes to the CMR examination, allowing CMR to provide comprehensive ventricular assessment. Thirdly, the data obtained are morphologically and quantitatively similar to that obtained using TTE, and are thus easily interpretable by a general cardiologist, requiring no further physician education for the interpretation of CMR results. The added ability to interrogate flow in 3D manner is an improvement to prior studies and has the potential to provide more accurate diastolic flow evaluation, given the great variety of physiologic conditions that can prevail, providing variables that include flow profiles and mean trans-valvular flow.

In conclusion, we have shown for the first time that there is good agreement between the CMR and TTE evaluation of diastolic function. The 3D CMR acquisition of one slice at the mitral valve leaflets provides reproducible and reliable flow data, without a time penalty compared to TTE, and is clinically feasible on current commercial CMR scanners.

## Abbreviations

CHF: Congestive heart failure; CMR: Cardiovascular magnetic resonance; TTE: Transthoracic echocardiography; PC: Phase contrast; LV: Left ventricle; MV: Mitral valve; PV: Pulmonary vein.

## Authors' contributions

VKR conceived and designed the study, analyzed data and drafted the manuscript. MD carried out the phantom experiments in MRI and helped draft the manuscript. JY and RBW operated the MRI scanner, organized and stored the data. KC participated in analyzing the MRI data. CT perfomed the transthoracic echocardiograms. DV performed the statistical analysis. RWWB participated in study design, coordination and scientific input. All authors read and approved the final manuscript.
